# Supplementary material for: Safety and Immunogenicity of the Tetravalent Recombinant COVID-19 Protein Vaccine SCTV01E in Children and Adolescents Aged 3 to 17 Years: A Randomized, Double-Blind, Placebo-Controlled Phase 2 Clinical Trial
Source: Vaccines (Basel). 2025 Jan 7;13(1):43. doi: 10.3390/vaccines13010043 (PMC11768740; doi:10.3390/vaccines13010043)
Supplement: Supplementary file 1 [file vaccines-13-00043-s001.zip › vaccines-3371582-supplementary.pdf]

Supplementary material

Table of Contents	Page
**1.Supplemental methods: Inclusion and exclusion criteria.**	2
**2.Supplemental methods: SARS-CoV-2 Omicron BA.5 live virus neutralization assay**	4
**3.Supplemental Table S1. Demographic characteristics of the participants in Group A (⩾18 years)**	5
**4. Supplemental Table S2. Details of solicited local AEs (SS)**	6
**5. Supplemental Table S3. Details of vaccine related solicited systemic AEs (SS)**	6
**6. Supplemental Table S4. Anti-Omicron BA.5 Neutralizing Antibody Test (live virus neutralizing test)**	7
**7. Supplemental Table S5. T Cell response of Group A (≥18 years)**	12

1. Supplemental methods: Inclusion and exclusion criteria

1.1 Inclusion criteria

Participants are eligible to be included in the study only if the following conditions are met:

1) Male or female aged ≥18 years old when signing ICF;
2) No previous anti-SARS-CoV-2 vaccine injected (licensed or investigational vaccine).
3) Participants can sign a written ICF and voluntarily participate in the study, and fully understand the procedure and risks of participating in the study;
4) Ability to read, understand, and fill in record cards;
5) Healthy participants or participants with stable underlying diseases. Stable underlying disease is defined as a condition that is stable for at least 3 months before enrollment in the study, with no significant change in treatment regimen and no hospitalization due to disease progression;
6) Fertile men and women of reproductive age voluntarily agree to use effective contraception from the time of signing the ICF until 6 months after full vaccination; Women of childbearing age had negative pregnancy tests during the screening period.

1.2 Exclusion criteria

A participant who conforms to any of the following criteria should not be enrolled in the study:

1) Presence of fever within 72 h before the study vaccination (axillary temperature ≥ 37.3℃) or in the active state of tuberculosis and other diseases;
2) A history of infection or disease related to severe acute respiratory syndrome (SARS), Middle East respiratory syndrome (MERS), or corresponding immunosuppressants;
3) A history of allergic reactions to any vaccine or drug, such as allergy, urticaria, severe skin eczema, dyspnea, laryngeal edema, and angioneurotic edema;
4) A medical or family history of seizure, epilepsy, encephalopathy, and psychosis;
5) Immunocompromised patients suffering from immunodeficiency diseases, important organ diseases, immune diseases (including Guillain-Barre Syndrome [GBS], systemic lupus erythematosus, rheumatoid arthritis, asplenia or splenectomy caused by any circumstances, and other immune diseases that may have an impact on immune response in the investigator's opinion), etc.;
6) Long-term use of immunosuppressant therapy or immunomodulatory drugs for ≥14 days within the first six months prior to enrollment, or plan to use immunosuppressant therapy or immunomodulatory drugs within two years after enrollment. Whereas short-term (≤14 days) use of oral, inhaled and topical steroids are allowed.
7) Phase I participants only: Previously or currently suffering from clinically significant cardiovascular diseases (except for the hypertension that can be controlled with drugs), or clinically significant disorders related to respiratory system, liver and kidney (except for light fatty liver), gastrointestinal system (except for chronic gastritis), endocrine system, blood and lymphatic system, metabolic and skeletal systems, or malignancies (except for skin basal cell carcinoma and carcinoma in-situ of cervix), that may affect the study assessment, or cause risks during the study vaccination, or interfere with the data interpretation as determined by the investigator;

8) Phase II participants only: Presence of severe or uncontrollable cardiovascular diseases, or severe or uncontrollable disorders related to endocrine system, blood and lymphatic system, liver and kidney, respiratory system, metabolic and skeletal systems, or malignancies (exception for skin basal cell carcinoma and carcinoma in-situ of cervix);
9) Contraindications for intramuscular injection or intravenous blood sampling, including thrombocytopenia and other blood coagulation disorders;
10) Participants who received any immunoglobulin or blood products in the previous 3 months, or plan to receive similar products during the study;
11) Participants who received other investigational drugs within 1 month before the study vaccination;
12) Participants vaccinated with influenza vaccine within 14 days or with other vaccines within 28 days before the study vaccination;
13) Those who donated blood or had blood loss (≥450 mL) within 3 months before the vaccination or plan to donate blood during the study period;
14) Those who are pregnant or breast-feeding;
15) Those who plan to donate ovum or sperms during the study period;
16) Those who cannot follow the study procedures, or cannot cooperate to complete the study due to planned relocation or long-term outing;
17) Those unsuitable for participating in the clinical study as determined by the investigator because of other abnormalities that are likely to confuse the study results, or non-conformance with the maximal benefits of the participants;
18) Phase I participants only: participants with active hepatitis B virus (HBV) infection (except asymptomatic carriers), hepatitis C virus (HCV), syphilis or HIV seropositive test;
19) Phase II participants only: those who are tested positive for HIV in terms of serology.

2. Supplemental methods: SARS-CoV-2 Omicron BA.5 live virus neutralization assay

The live virus neutralization assays were performed by National Institute for Viral Disease Control and Prevention of Chinese Center for Disease Control and Prevention. The Serum samples were heat inactivated for 30 minutes at 56 ºC and eight two-fold serial dilutions of test samples were prepared in a separate dilution plate with the first dilution being 1:4. The 50μl diluted sera were mixed with an equal volume of SARS-CoV-2 Omicron BA.5 variants and incubated for 1~2 hour at 37°C, 5% CO_2. The virus/serum mixtures were then transferred to sub-confluent Vero E6 cell monolayer plates (E6 cells were pre-seeded 24 hours beforehand). Plates were incubated for 3-5 days at 37°C, 5% CO_2. The residual non-neutralized virus was detected via cytopathic effect (CPE) by microscopic scoring. The neutralization titers were expressed as the reciprocal of the highest dilution at which 50% of the replicate wells were protected from infection (PRNT50).

3. Supplemental Table S1. Demographic characteristics of the participants in Group A (⩾18 years)

Group A (≥18 years)	Placebo (N = 50)	SCTV01E (N = 200)	Totao (N = 250)
**Age (years)**			
N	50	200	250
Mean (SD)	37.8 (10.31)	38.3 (9.25)	38.2 (9.46)
Median (Range)	36.5 (18, 59)	36.0 (18, 58)	36.0 (18, 59)
**Gender - n (%)**			
Male	16 (32.0)	75 (37.5)	91 (36.4)
Female	34 (68.0)	125 (62.5)	159 (63.6)
**Ethnic - n (%)**			
Han Chinese	49 (98.0)	200 (100.0)	249 (99.6)
Other Chinese	1 (2.0)	0	1 (0.4)
**Height (cm)**			
N	50	200	250
Mean (SD)	161.1 (9.18)	161.6 (7.62)	161.5 (7.94)
Median (Range)	161.0 (145, 184)	161.0 (141, 182)	161.0 (141, 184)
**Weight (kg)**			
N	50	200	250
Mean (SD)	59.33 (11.390)	61.79 (10.312)	61.30 (10.559)
Median (Range)	57.20 (41.7, 89.4)	61.30 (42.2, 106.4)	60.85 (41.7,106.4)
**BMI (kg/m^2)**			
N	50	200	250
Mean (SD)	22.74 (3.031)	23.60 (2.978)	23.42 (3.003)
Median (Range)	22.65 (17.3, 29.9)	23.55 (16.9, 34.3)	23.30 (16.9, 34.3)
**COVID-19 Vaccination History**			
Primary	3 (6.0)	17 (8.5)	20 (8.0)
Booster	47 (94.0)	183 (91.5)	230 (92.0)
**Last COVID-19 Vaccination**			
Inactivated	47 (94.0)	187 (93.5)	234 (93.6)
Non-inactivated	3 (6.0)	13 (6.5)	16 (6.4)
**Vaccination Time Interval**			
6~12 months	4 (8.0)	16 (8.0)	20 (8.0)
12~24 months	46 (92.0)	184 (92.0)	230 (92.0)

SD: Standard Deviation

BMI: Body Mass Index

4. Supplemental Table S2. Details of solicited local AEs (SS)

Groups	Placebo	SCTV01E
**Group A (≥18 years)**	**N = 50**	**N = 200**
**At least one solicited local AE**	**2 (4.0)**	**67 (33.5)**
Injection site pain	2 (4.0)	64 (32.0)
Injection site swelling	0	5 (2.5)
Injection site pruritus	0	4 (2.0)
Injection site induration	0	3 (1.5)
Injection site erythema	0	2 (1.0)
**Group B (12-17 years)**	**N = 16**	**N = 63**
**At least one solicited local AE**	**0**	**16 (25.4)**
Injection site pain	0	14 (22.2)
Injection site swelling	0	5 (7.9)
Injection site erythema	0	4 (6.3)
Injection site induration	0	1 (1.6)
**Group C (3-11 years)**	**N = 38**	**N = 151**
**At least one solicited local AE**	**1 (2.6)**	**40 (26.5)**
Injection site pain	1 (2.6)	35 (23.2)
Injection site swelling	0	12 (7.9)
Injection site erythema	0	8 (5.3)
Injection site induration	0	6 (4.0)
Injection site pruritus	0	4 (2.6)
Injection site rash	0	2 (1.3)

AE: Adverse Event

All solicited local AEs were considered to be related to vaccine.

5. Supplemental Table S3. Details of vaccine related solicited systemic AEs (SS)

Groups	Placebo	SCTV01E
**Group A (≥18 years)**	**N = 50**	**N = 200**
**At least one solicited systemic AE**	**2 (4.0)**	**28 (14.0)**
Fever	1 (2.0)	11 (5.5)
Fatigue	1 (2.0)	9 (4.5)
Headache	1 (2.0)	6 (3.0)
Cough	1 (2.0)	5 (2.5)
Myodynia	0	5 (2.5)
Arthralgia	0	3 (1.5)
Nausea	0	1 (0.5)
Anxiety	0	1 (0.5)
**Group B (12-17 years)**	**N = 16**	**N = 63**
**At least one solicited systemic AE**	**0**	**18 (28.6)**
Fatigue	0	13 (20.6)
Fever	0	12 (19.0)
Headache	0	9 (14.3)

Groups	Placebo	SCTV01E
Myodynia	0	5 (7.9)
Arthralgia	0	3 (4.8)
Cough	0	3 (4.8)
Nausea	0	2 (3.2)
Constipation	0	1 (1.6)
Insomnia	0	1 (1.6)
Chill	0	1 (1.6)
Pruritus	0	1 (1.6)
**Group C (3-11 years)**	**N = 38**	**N = 151**
**At least one solicited systemic AE**	**4 (10.5)**	**66 (43.7)**
Fever	1 (2.6)	57 (37.7)
Headache	1 (2.6)	20 (13.2)
Cough	0	13 (8.6)
Fatigue	0	9 (6.0)
Vomiting	0	6 (4.0)
Chill	0	6 (4.0)
Nausea	1 (2.6)	4 (2.6)
Myodynia	1 (2.6)	1 (0.7)
Constipation	0	1 (0.7)
Arthralgia	0	1 (0.7)
Syncope	0	1 (0.7)

AE: Adverse Event

6. Supplemental Table S4. Anti-Omicron BA.5 Neutralizing Antibody Test (live virus neutralizing test)

6.1 GMT of anti-Omicron BA.5 Neutralizing Antibody on days 28, 90 and 180 (I-PPS)

Groups	Placebo	SCTV01E
**Group A (⩾18 years)**	**N = 50**	**N = 200**
**Baseline (Day 0)**		
N	50	200
GMT (95% CI)	228 (163, 317)	253 (218, 293)
**Day 28**		
n	49	200
GMT (95% CI)	115 (90, 147)	465 (411, 525)
GMFI (95% CI)	0.5 (0.40, 0.68)	1.8 (1.54, 2.19)
LS GMR (95% CI) [1]		3.94 (3.03, 5.14)
P [1]		<0.001
**Day 90**		
n	48	197
GMT (95% CI)	145 (111, 189)	442 (396, 493)
GMFI (95% CI)	0.7 (0.52, 0.89)	1.8 (1.48, 2.08)
LS GMR (95% CI) [1]		2.95 (2.30, 3.78)

Groups	Placebo	SCTV01E
P [1]		<0.001
**Day 180**		
n	47	192
GMT (95% CI)	127 (96, 169)	347 (312, 387)
GMFI (95% CI)	0.6 (0.41, 0.89)	1.4 (1.17, 1.68)
LS GMR (95% CI) [1]		2.72 (2.11, 3.51)
P [1]		<0.001
**Group B (12-17 years)**	**N = 15**	**N = 56**
**Baseline (Day 0)**		
n	15	56
GMT (95% CI)	90 (29, 277)	67 (37, 119)
**Day 28**		
n	14	50
GMT (95% CI)	72 (23, 227)	1314 (1002, 1724)
GMFI (95% CI)	0.9 (0.56, 1.46)	19.3 (10.54, 35.36)
LS GMR (95% CI) [1]		18.48 (9.43, 36.24)
P [1]		<0.001
**Day 90**		
n	9	37
GMT (95% CI)	75 (17, 320)	1146 (820, 1601)
GMFI (95% CI)	1.2 (0.23, 5.84)	17.6 (7.58, 40.74)
LS GMR (95% CI) [1]		15.33 (6.11, 38.47)
P [1]		<0.001
**Day 180**		
n	10	33
GMT (95% CI)	62 (20, 195)	1149 (721, 1833)
GMFI (95% CI)	0.7 (0.17, 2.68)	11.9 (4.52, 31.44)
LS GMR (95% CI) [1]		18.65 (6.72, 51.75)
P [1]		<0.001
**Group C (3-11 years)**	**N = 35**	**N = 135**
**Baseline (Day 0)**		
n	35	135
GMT (95% CI)	45 (21, 95)	50 (34, 72)
**Day 28**		
n	35	135
GMT (95% CI)	68 (32, 146)	1668 (1447, 1922)
GMFI (95% CI)	1.5 (0.97, 2.36)	33.5 (23.56, 47.70)
LS GMR (95% CI) [1]		24.20 (16.08, 36.42)
P [1]		<0.001
**Day 90**		
n	26	96
GMT (95% CI)	137 (64, 293)	1459 (1262, 1685)
GMFI (95% CI)	2.0 (1.00, 4.14)	24.4 (14.83, 40.22)
LS GMR (95% CI) [1]		10.81 (6.73, 17.36)

Groups	Placebo	SCTV01E
P [1]		<0.001
**Day 180**		
n	25	104
GMT (95% CI)	100 (55, 181)	899 (769, 1051)
GMFI (95% CI)	2.2 (0.98, 5.11)	17.0 (10.20, 28.29)
LS GMR (95% CI) [1]		9.27 (6.08, 14.12)
P [1]		<0.001

GMT: Geometric mean titers;

GMFI: Geometric mean fold increase;

LS GMR: Least square Geometric Mean Ratio;

[1] Based on the LOG-transformed Analysis of Covariance model (ANCOVA), covariables include trial groups, random stratification factors, and LOG-transformed baseline values.

6.2 SRR of anti-Omicron BA.5 Neutralizing Antibody on days 28, 90 and 180 (I-PPS)

Groups	Placebo	SCTV01E
**Group A (⩾18 years)**	**N = 50**	**N = 200**
**Day 28**		
SRR，n/N	0.0 (0/49)	24.5 (49/200)
SRR (95% CI) [1]	0.00, 7.25	18.71, 31.06
SRR difference (95% CI) [2]		24.75 (18.63, 30.86)
P [2]		<0.001
**Day 90**		
SRR，n/N	4.2 (2/48)	20.3 (40/197)
SRR (95% CI) [1]	0.51, 14.25	14.92, 26.61
SRR difference (95% CI) [2]		16.05 (8.01, 24.08)
P [2]		0.008
**Day 180**		
SRR，n/N	6.4 (3/47)	23.4 (45/192)
SRR (95% CI) [1]	1.34, 17.54	17.64, 30.08
SRR difference (95% CI) [2]		17.98 (8.79, 27.17)
P [2]		0.007
**Group B (12-17 years)**	**N = 15**	**N = 56**
**Day 28**		
SRR，n/N	7.1 (1/14)	74.0 (37/50)
SRR (95% CI) [1]	0.18, 33.87	59.66, 85.37
SRR difference (95% CI) [2]		66.33 (48.03, 84.62)
P [2]		<0.001
**Day 90**		
SRR，n/N	22.2 (2/9)	70.3 (26/37)
SRR (95% CI) [1]	2.81, 60.01	53.02, 84.13
SRR difference (95% CI) [2]		48.05 (17.15, 78.95)

Groups	Placebo	SCTV01E
P [2]		0.009
**Day 180**		
SRR，n/N	20.0 (2/10)	54.5 (18/33)
SRR (95% CI) [1]	2.52, 55.61	36.35, 71.89
SRR difference (95% CI) [2]		34.55 (4.49, 64.60)
P [2]		0.058
**Group C (3-11 years)**	**N = 35**	**N = 135**
**Day 28**		
SRR，n/N	17.1 (6/35)	86.7 (117/135)
SRR (95% CI) [1]	6.56, 33.65	79.75, 91.90
SRR difference (95% CI) [2]		70.10 (56.58, 83.62)
P [2]		<0.001
**Day 90**		
SRR，n/N	38.5 (10/26)	71.9 (69/96)
SRR (95% CI) [1]	20.23, 59.43	61.78, 80.58
SRR difference (95% CI) [2]		34.33 (13.71, 54.95)
P [2]		0.001
**Day 180**		
SRR，n/N	36.0 (9/25)	59.6 (62/104)
SRR (95% CI) [1]	17.97, 57.48	49.54, 69.13
SRR difference (95% CI) [2]		25.42 (4.17, 46.68)
P [2]		0.025

SRR: Sero-Response Rate;

[1] Clopper-Pearson exact confidence interval;

[2] Based on the Cochran - Mantel - Haenszel test (CMH) method, stratification factors are taken as covariables.

6.3 GMT of anti-Omicron BA.5 Neutralizing Antibody on days 7 and 14 in Group A and B (I-PPS)

Groups	Placebo	SCTV01E
**Group A (⩾18 years)**	**N = 50**	**N = 200**
**Baseline (Day 0)**		
n	50	200
GMT (95% CI)	228 (163, 317)	253 (218, 293)
**Day 7**		
N	24	101
GMT (95% CI)	134 (79, 227)	345 (304, 392)
GMFI (95% CI)	0.9 (0.68, 1.11)	1.7 (1.41, 2.01)
LS GMR (95% CI) [1]		2.29 (1.70, 3.08)
P [1]		<0.001
**Day 14**		
n	23	101
GMT (95% CI)	105 (66, 167)	363 (322, 410)

Groups	Placebo	SCTV01E
GMFI (95% CI)	0.7 (0.56, 0.84)	1.8 (1.45, 2.16)
LS GMR (95% CI) [1]		3.17 (2.36, 4.27)
P [1]		<0.001
**Group B (12-17 years)**	**N = 15**	**N = 56**
**Baseline (Day 0)**		
n	15	56
GMT (95% CI)	90 (29, 277)	67 (37, 119)
**Day 7**		
n	14	55
GMT (95% CI)	76 (22, 264)	484 (339, 691)
GMFI (95% CI)	0.8 (0.58, 1.16)	7.3 (4.68, 11.47)
LS GMR (95% CI) [1]		7.52 (3.95, 14.31)
P [1]		<0.001
**Day 14**		
n	12	43
GMT (95% CI)	60 (17, 213)	1128 (883, 1440)
GMFI (95% CI)	0.7 (0.33, 1.35)	16.3 (8.57, 30.83)
LS GMR (95% CI) [1]		19.95 (10.21, 38.99)
P [1]		<0.001

GMT: Geometric mean titers;

GMFI: Geometric mean fold increase;

LS GMR: Least square Geometric Mean Ratio;

[1] Based on the LOG-transformed Analysis of Covariance model (ANCOVA), covariables include trial groups, random stratification factors, and LOG-transformed baseline values.

6.4 SRR of anti-Omicron BA.5 Neutralizing Antibody on days 7 and 14 in Group A and B （I-PPS）

Groups	Placebo	SCTV01E
**Group A (≥18 years)**	**N = 50**	**N = 200**
**Day 7**		
SRR，n/N	0.0 (0/24)	12.9 (13/101)
SRR (95% CI) [1]	0.00, 14.25	7.04, 21.00
SRR difference (95% CI) [2]		13.43 (6.59, 20.26)
P [2]		0.056
**Day 14**		
SRR，n/N	0.0 (0/23)	15.8 (16/101)
SRR (95% CI) [1]	0.00, 14.82	9.33, 24.45
SRR difference (95% CI) [2]		16.21 (8.81, 23.62)
P [2]		0.042
**Group B (12-17 years)**	**N = 15**	**N = 56**
**Day 7**		
SRR，n/N	0.0 (0/14)	63.6 (35/55)

SRR (95% CI) [1]	0.00, 23.16	49.56, 76.19
SRR difference (95% CI) [2]		62.96 (50.08, 75.84)
P 2]		<0.001
**Day 14**		
SRR，n/N	0.0 (0/12)	69.8 (30/43)
SRR (95% CI) [1]	0.00, 26.46	53.87, 82.82
SRR difference (95% CI) [2]		69.77 (56.04, 83.49)
P [2]		<0.001

SRR: Sero-Response Rate;

[1] Clopper-Pearson exact confidence interval;

[2] Based on the Cochran - Mantel - Haenszel test (CMH) method, stratification factors are taken as covariables.

7. Supplemental Table S5. T Cell response of Group A (≥18 years)

7.1 Number of IFN-γ secreting T cell (FAS)

	Value		Change compared with baseline	
	**Placebo**	**SCTV01E**	**Placebo**	**SCTV01E**
**Baseline (Day 0)**				
N	13	62		
Mean (SD)	28.9 (23.16)	44.4 (37.95)		
Median (Range)	28.0 (0, 72)	36.0 (0, 168)		
**Day 7**				
n	13	62	13	62
Mean (SD)	32.6 (23.03)	110.9 (69.26)	3.7 (25.74)	66.5 (73.29)
Median (Range)	24.0 (0, 76)	92.0 (0, 352)	0.0 (-24, 60)	52.0 (-104, 312)
P [1]		<0.001		<0.001
**Day 90**				
n	12	60	12	60
Mean (SD)	29.0 (46.65)	33.0 (23.84)	-0.3 (34.17)	-9.9 (39.20)
Median (Range)	14.0 (4, 172)	28.0 (0, 96)	-12.0 (-28, 100)	-4.0 (-164, 80)
P [1]		0.044		0.695

[1] The Mann-Whitney U test was used.

7.2 Number of IL-4 secreting T cell (FAS)

	Value		Change compared with baseline	
	Placebo	SCTV01E	Placebo	SCTV01E
**Baseline (Day 0)**				
n	13	62		
Mean (SD)	16.9 (22.93)	27.9 (51.55)		
Median (Range)	8.0 (0, 68)	10.0 (0, 344)		
**Day 7**				
n	13	62	13	62
Mean (SD)	12.3 (13.31)	122.1 (90.81)	-4.6 (16.96)	94.2 (84.92)

	Value		Change compared with baseline	
	Placebo	SCTV01E	Placebo	SCTV01E
Median (Range)	4.0 (0, 32)	108.0 (12, 376)	0.0 (-44, 24)	90.0 (-52, 364)
P [1]		<0.001		<0.001
**Day 90**				
n	12	60	12	60
Mean (SD)	9.0 (17.82)	23.5 (29.23)	-3.7 (18.41)	-5.1 (38.76)
Median (Range)	4.0 (0, 64)	16.0 (0, 172)	-4.0 (-48, 36)	0.0 (-172, 104)
P [1]		0.006		0.497

[1] The Mann-Whitney U test was used.
